# Robust unmanned aerial vehicles tracking amid electronic interference utilizing auxiliary particle filtering

**DOI:** 10.1371/journal.pone.0333009

**Published:** 2025-09-29

**Authors:** Le Qi, Tao Zhang, Guoming Chen, Wanyang Wang

**Affiliations:** Aviation Maintenance NCO School, Air Force Engineering University, Xinyang, Henan, China; Buckinghamshire New University - High Wycombe Campus: Buckinghamshire New University, UNITED KINGDOM OF GREAT BRITAIN AND NORTHERN IRELAND

## Abstract

Electronic interference poses a significant challenge to Unmanned Aerial Vehicle (UAV) tracking systems, compromising navigation accuracy and operational safety in critical applications such as surveillance, disaster response, and infrastructure inspection. This study introduces a novel application of the Auxiliary Particle Filter (APF) for robust UAV tracking under interference conditions, focusing on fixed-reference scenarios. The APF incorporates adaptive proposal distributions and robust weight updates to effectively mitigate interference-induced measurement errors. Through comprehensive simulation that evaluates performance under varying interference and sensor degradation scenarios, the APF demonstrates superior accuracy, achieving a mean Root Mean Square Error (RMSE) of 4.82 meters with low variability (σ=0.30m). This significantly outperforms traditional filters, including the Extended Kalman Filter (EKF) and Unscented Kalman Filter (UKF). Notably, the APF maintains stable performance even under severe interference conditions, where conventional approaches exhibit substantial degradation. Statistical validation confirms these improvements across all test scenarios (*p* < 0.001). The detailed implementation guidelines provided in this study enable adoption across diverse operational contexts. These findings establish the APF as a robust and reliable solution for interference-prone environments and lay the groundwork for its adaptation to more dynamic tracking scenarios in autonomous UAV operations.

## Introduction

Unmanned Aerial Vehicles (UAVs) have revolutionized numerous sectors, including surveillance, environmental monitoring, disaster management, and logistics, owing to their flexibility and operational efficiency [1]. Central to the efficacy of UAVs in these applications is their ability to maintain precise navigation and tracking capabilities. Accurate state estimation ensures reliable operation, preventing mishaps such as collisions, loss of control, and mission failures [2].

However, UAV tracking systems are vulnerable to various perturbations, notably electronic interference from jamming devices, environmental factors, and overlapping communication frequencies [3]. Such interference can corrupt sensor measurements, leading to inaccurate estimations of position, velocity, and orientation. Traditional filtering methods like the Extended Kalman Filter (EKF) and Unscented Kalman Filter (UKF), while computationally efficient, often falter under significant non-linearities and non-Gaussian noise introduced by electronic interference [4]. This is particularly challenging in UAV networks where robustness to interference and faults is critical [5].

Particle Filters (PFs), leveraging sequential Monte Carlo methods, offer a robust alternative for handling non-linear and non-Gaussian systems [6]. Nevertheless, standard PFs are susceptible to particle degeneracy and sample impoverishment, especially in high-dimensional spaces or with limited particle numbers [7]. The Auxiliary Particle Filter (APF) enhances the standard PF by incorporating an auxiliary variable, thereby improving the proposal distribution and mitigating weight variance [8].

This study focuses on UAV tracking relative to a fixed reference point, such as a stationary base station or landmark. This scenario is prevalent in controlled environments where UAVs must maintain precise positions relative to known static locations. By isolating the tracking problem to a fixed reference point, we can effectively evaluate the APF’s performance under electronic interference without the complexities introduced by dynamic target movements. This foundational understanding paves the way for extending the filter’s applicability to more dynamic scenarios in future research.

The key contributions of this work include:

- A systematic analysis of APF performance in UAV tracking under various electronic interference conditions, with specific focus on fixed-reference scenarios. While APF has been studied in various tracking contexts, its application to UAV tracking under electronic interference remains unexplored.
- Development of a comprehensive simulation framework enabling systematic comparison of different filtering approaches (EKF, UKF, PF, APF, and RBPF) under controlled interference conditions. Our framework provides:
  - Detailed tracking accuracy analysis under varying interference conditions
  - Computational efficiency comparisons
  - Robustness evaluation in challenging scenarios
- Practical implementation guidelines for real-time UAV tracking, including parameter selection strategies, state estimation procedures under interference, and performance optimization techniques.

The remainder of this paper is structured as follows: Section “Related work” reviews related literature on UAV tracking and filtering methods, including a comprehensive comparison of existing filtering approaches. Section “Materials and methods” presents our methodology, detailing the problem formulation, stochastic system dynamics, Monte Carlo simulation framework, and the proposed Auxiliary Particle Filter design. Section “Results” presents a comprehensive performance analysis, including baseline metrics, interference sensitivity, statistical validation, APF characteristics, comparative filter performance, implementation considerations, and identified limitations. Finally, Section “Discussion” concludes the paper with a summary of key contributions and outlines future research directions, including our planned real-world validation program.

## Related work

UAV tracking and navigation have been the subject of extensive research, with numerous studies focusing on improving state estimation accuracy under various operational challenges. Traditional filtering methods, such as the Extended Kalman Filter (EKF), have been widely adopted for UAV state estimation due to their computational efficiency and ability to manage mild non-linearities [9]. However, EKFs inherently rely on linear approximations of non-linear systems, which can result in significant estimation errors in environments characterized by strong non-linearities and non-Gaussian noise [10]. Recent comprehensive studies have demonstrated the necessity of robust control strategies for UAVs operating under constrained conditions. Kong *et al*. [11] established that effective state estimation is crucial for maintaining control performance, particularly when dealing with windowed output constraints in VTOL-UAVs. Their findings emphasize the importance of developing filtering approaches that can maintain accuracy under operational constraints, which aligns with our focus on electronic interference conditions.

The Unscented Kalman Filter (UKF) was developed to address some of the EKF’s limitations by utilizing sigma points to better capture the mean and covariance of non-linear transformations [12]. While the UKF improves accuracy in certain scenarios, it may still struggle under severe non-linearities and non-Gaussian noise, particularly in the presence of electronic interference [13].

Particle Filters (PFs) offer a more flexible framework for state estimation, capable of managing highly non-linear and non-Gaussian systems through sequential Monte Carlo methods [14]. PFs have shown enhanced performance in UAV tracking within complex environments [15]. However, their practical implementation faces significant challenges. Li *et al*. [16] provide a comprehensive analysis of these challenges, particularly highlighting sample degeneracy and impoverishment as critical issues in particle filtering. Their systematic review of intelligent approaches to combat these limitations has established a foundation for developing more robust particle filtering implementations. Despite these methodological advances, standard PFs still struggle with maintaining particle diversity and effective state estimation in high-dimensional spaces or when computational resources limit particle counts.

Recent advances in UAV systems have explored various robust statistical approaches to address operational challenges. Notably, Liu *et al*. [17] demonstrated the effectiveness of *t*-distributions in evolutionary optimization for UAV path planning, achieving improved robustness in complex geographical environments through adaptive probability adjustments. While their work established the utility of *t*-distributions in UAV applications, it focused primarily on path optimization rather than state estimation. Our work represents a fundamental shift in the application of *t*-distributions, leveraging their robust properties for weight updates in particle filtering to address the distinct challenge of electronic interference in tracking applications.

To address these challenges, the Auxiliary Particle Filter (APF) was introduced, incorporating an auxiliary variable to improve the proposal distribution and maintain particle diversity. Recent implementations have demonstrated the APF’s robustness in complex tracking scenarios. Particularly noteworthy is the work of Razavi *et al*. [18], who achieved significant performance improvements in challenging indoor environments by combining APF with deep learning techniques. Their results demonstrated superior tracking accuracy and resilience to measurement uncertainties, suggesting promising applications for UAV tracking under interference conditions. The APF addresses key limitations of standard particle filters by incorporating auxiliary variables to improve proposal distribution quality and maintain particle diversity during resampling. Despite these encouraging advances in related domains, the specific application of APF to UAV tracking under electronic interference remains underexplored, presenting an opportunity for significant contributions to the field.

Recent developments in UAV tracking under adverse conditions have further highlighted the importance of robust filtering approaches. Gabr *et al*. [19] introduced SMART-TRACK, a novel Kalman filter-guided sensor fusion approach for robust UAV object tracking in dynamic environments, demonstrating significant improvements in tracking accuracy through high-frequency state estimates and adaptive measurement re-acquisition. Similarly, Zhu *et al*. [20] proposed RNN-enhanced IMM-KF algorithms for UAV trajectory tracking using ADS-B data, achieving a 28.56% reduction in root mean square error compared to traditional IMM-KF methods by adaptively adjusting noise matrices through neural network assistance. Almasoud *et al*. [21] developed robust anti-jamming techniques for UAV data collection in IoT networks using landing platforms and reconfigurable intelligent surfaces, addressing the critical challenge of maintaining communication integrity in the presence of malicious jammers. Liu *et al*. [22] extended these approaches by implementing jamming-enhanced secure UAV communications with propulsion energy and curvature radius constraints, demonstrating enhanced resilience against eavesdropping attacks through optimized trajectory and power allocation strategies. Additionally, Xing *et al*. [23] proposed a deep reinforcement learning approach for secure UAV communications under jamming conditions, achieving robust state estimation through intelligent adaptive anti-jamming mechanisms. These recent advances collectively demonstrate the growing recognition of interference mitigation as a critical challenge in UAV tracking systems and establish the foundation for more sophisticated approaches like the APF methodology investigated in this study.

Rao-Blackwellized Particle Filters (RBPFs) represent another development in this field, decomposing the state into linear and non-linear components to reduce estimation variance through marginalization. This approach has been demonstrated in sensor fusion applications [24]. However, their effectiveness in scenarios involving severe electronic interference and sensor degradation, as in UAV tracking, remains largely unexplored.

Building upon these foundational works, this paper investigates the application of APF to UAV tracking under electronic interference. As summarized in Table 1, each filtering approach has distinct advantages and limitations. Our work provides a systematic comparison of APF against EKF, UKF, PF, and RBPF in a realistic simulation framework, specifically focusing on performance under electronic interference conditions. This comparative analysis aims to provide insights into the relative strengths and limitations of each filtering method in challenging operational environments.

**Table 1:** Characteristics of different filtering approaches in UAV state estimation. Methods based on [25,12,8], and [24].

Method	Key Strength	Main Limitation
EKF [25]	Computational efficiency	Limited to mild non-linearities
UKF [12]	Better non-linear handling	Struggles with non-Gaussian noise
Standard PF [8]	Handles non-linear scenarios well	Particle degeneracy issues
RBPF [24]	Reduced estimation variance	Computational complexity

## Materials and methods

This section presents a comprehensive framework for robust UAV tracking under electronic interference conditions. We first introduce the theoretical foundations and system architecture, followed by detailed formulations of individual components and their interconnections within a rigorous Monte Carlo evaluation framework.

### Problem formulation and system architecture

**Fig 1:**
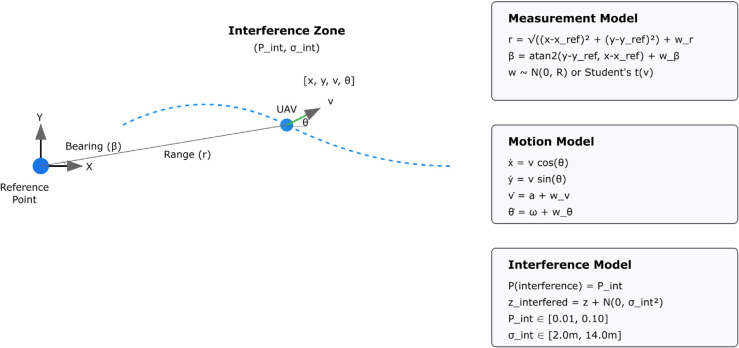
UAV tracking control schematic. System architecture showing sensor placement, coordinate systems, and interference sources affecting range-bearing measurements in planar tracking scenarios.

While UAV tracking has been extensively studied, the presence of electronic interference presents unique challenges that traditional approaches struggle to address. Our framework specifically addresses three key challenges:

(1) the non-Gaussian nature of interference-induced measurement errors,

(2) the intermittent nature of interference events, and

(3) the need for robust state estimation under sensor degradation. The proposed architecture integrates these considerations through:

Fig 1 illustrates the UAV tracking control schematic showing the geometric relationships, measurement models, and interference characteristics. The system maintains state estimation relative to a fixed reference point under stochastic interference conditions, with explicit modeling of range-bearing measurements and motion dynamics.

#### State space representation.

As depicted in Fig 1, we consider a planar UAV tracking scenario characterized by the state vector $𝐱=[x,y,v,\theta {]}^{T}\in {\mathbb{R}}^{4}$, where (*x*,*y*) denotes the UAV position in Cartesian coordinates, *v* represents scalar velocity magnitude, and *θ* indicates heading angle. The state evolution follows a nonlinear stochastic differential equation [11]:

d𝐱=f(𝐱,t)dt+G(𝐱)d𝐰 (1)

where f(·) represents the nonlinear drift term, G(·) is the diffusion matrix, and d𝐰 is a Wiener process with covariance *Q*.

#### Measurement and interference model.

Traditional measurement models typically assume Gaussian noise, which fails to capture the heavy-tailed error distributions characteristic of electronic interference. Our model innovates by explicitly separating standard sensor noise from interference effects:

$$z_{k}=h({𝐱}_{k})+v_{k}+\eta_{k}I_{k}$$ (2)

$$h(𝐱)=[\begin{array}{c} \sqrt{x^{2}+y^{2}}\\ \arctan2(y,x) \end{array}]$$ (3)

where $h(\cdot ):{\mathbb{R}}^{4}\to {\mathbb{R}}^{2}$ is the nonlinear measurement function mapping states to range-bearing observations, $v_{k}\sim 𝒩(0,R)$ represents measurement noise, $\eta_{k}\sim 𝒩(0,\sigma_{\text{int}}^{2})$ denotes interference magnitude when present, $\sigma_{\text{int}}\in {\mathbb{R}}^{+}$ represents the standard deviation of interference magnitude, and $I_{k}\sim \text{Bernoulli}(P_{\text{int}})$ is the interference indicator defined on probability space (Ω,ℱ,P) where Ω is the sample space of interference events, ℱ is the associated *σ*-algebra, and *P* is the probability measure with $P(I_{k}=1)=P_{\text{int}}$. The random variables *I*_*k*_ and $\eta_{k}$ are stochastically independent [26].

This decomposition enables the filter to adaptively handle both routine measurement uncertainty and severe interference events through different mechanisms, improving robustness compared to single-noise models used in previous work.

### Stochastic system dynamics

#### Continuous-time motion model.

The UAV dynamics are governed by a set of coupled stochastic differential equations:

$$dx=v\cos(\theta )dt+\sigma_{x}dW_{x}\;dy=v\sin(\theta )dt+\sigma_{y}dW_{y}\;dv=[a_{0}\sin(\omega t)]dt+\sigma_{v}dW_{v}\;d\theta =[\omega_{0}\sin(\omega t)]dt+\sigma_{\theta }dW_{\theta }$$ (4)

where *a*_0_ = 0.05 and $\omega_{0}=2^{\circ }$ represent nominal acceleration and turn rate amplitudes, ω=0.01 is the oscillation frequency, and *W*_*i*_ are independent Wiener processes.

#### Discretized implementation.

For numerical implementation with time step *dt*, we employ a modified Euler-Maruyama scheme:

$${𝐱}_{k+1}={𝐱}_{k}+f({𝐱}_{k},k\cdot dt)dt+G({𝐱}_{k})\sqrt{dt}\xi_{k}$$ (5)

where $\xi_{k}\sim 𝒩(0,Q)$ represents the discretized Wiener increment with covariance $Q=\text{diag}(1.0,1.0,0.5,(\pi /180){\;}^{2})$. The relationship between continuous and discrete noise terms follows the standard discretization property where the discrete noise variance scales with $\sqrt{dt}$ to maintain consistent statistical properties across different time step sizes, ensuring that $𝔼[\xi_{k}\xi_{k}^{T}]=Q$ preserves the continuous-time stochastic differential equation characteristics. The diagonal elements represent variances in position (m^2^), velocity (m^2^/s^2^), and heading (rad^2^) respectively.

### Monte Carlo simulation framework

While Monte Carlo simulation is a standard evaluation approach, our framework introduces several key innovations:

1) A structured interference generation process that realistically models both the sporadic nature of interference events and their varying intensity.

2) Comprehensive performance evaluation that goes beyond simple RMSE to assess filter robustness under different interference patterns.

3) Statistical validation designed to specifically quantify improvements in handling interference-induced outliers.

**Fig 2:**
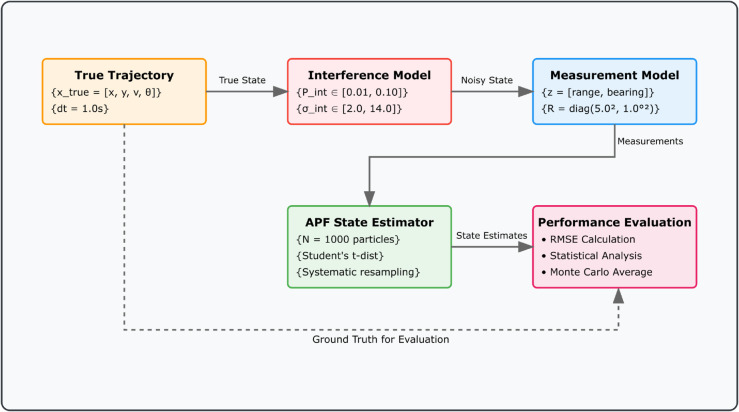
Comprehensive system architecture. Processing pipeline from trajectory generation through Monte Carlo evaluation, highlighting modular design for systematic filter comparison under controlled interference scenarios.

Fig 2 shows the comprehensive system architecture illustrating the integration of trajectory generation, interference modeling, state estimation, and performance evaluation within the Monte Carlo simulation framework. The modular design enables systematic evaluation of filter performance under varying interference conditions.

The evaluation framework, illustrated in Fig 2, implements a structured Monte Carlo simulation with *M* = 30 independent runs to ensure statistical robustness. Each run incorporates multiple interconnected components:

#### True trajectory generation.

The framework generates true state trajectories according to:

$${𝐱}_{\text{true}}=[x,y,v,\theta {]}^{T},\;dt=1.0s$$ (6)

where state evolution follows the continuous-time motion model described in Section “Stochastic system dynamics”.

#### Interference generation.

The interference model employs a compound stochastic process:

$$P(I_{k}=1)=P_{\text{int}}\in \{0.01,0.03,0.05,0.07,0.10\}\;\eta_{k}|I_{k}\sim 𝒩(0,\sigma_{\text{int}}^{2}),\;\sigma_{\text{int}}\in \{2.0,5.0,8.0,11.0,14.0\}\;S_{k}\sim \text{Bernoulli}(1-P_{\text{failure}})$$ (7)

where *S*_*k*_ represents sensor availability with $P_{\text{failure}}=0.05$.

#### Measurement generation.

The measurement model produces range-bearing observations:

$${𝐳}_{k}=[r_{k},\beta_{k}{]}^{T}+{𝐯}_{k}+\eta_{k}I_{k}1_{2}$$ (8)

where $1_{2}$ is a two-dimensional vector of ones, representing uniform interference effects on both range and bearing measurements, and with measurement noise covariance:

$$R=\text{diag}(5.0^{2},(1.0^{\circ }{)}^{2})$$ (9)

#### State estimation.

The APF state estimator maintains:

- Particle set: *N* = 1000 particles
- Weight update using Student’s *t*-distribution
- Systematic resampling when $N_{\text{eff}}<0.6N$

#### Performance evaluation.

Each Monte Carlo run evaluates:

$${\text{RMSE}}_{j}=\sqrt{\frac{1}{K}\sum_{k=1}^{K}\|{𝐱}_{j,k}-{\hat{𝐱}}_{j,k}{\|}^{2}}$$ (10)

The final performance metrics are computed by averaging across all *M* runs:

$${\text{RMSE}}_{\text{total}}=\frac{1}{M}\sum_{j=1}^{M}{\text{RMSE}}_{j}$$ (11)

This comprehensive framework enables systematic evaluation of filter performance under varying interference conditions while maintaining reproducibility through structured parameter variations and statistical analysis.

### Auxiliary particle filter design

The choice of APF as the core filtering algorithm is motivated by three key theoretical advantages for interference scenarios:

1) The auxiliary variable mechanism enables more informed particle proposal under interference by incorporating current measurements into the sampling process, addressing a key limitation of standard particle filters.

2) The Student’s *t*-distribution in the weight update provides natural robustness to outliers caused by interference, without requiring explicit outlier detection or measurement gating.

3) The adaptive degrees of freedom ($\nu_{k}$) allows the filter to automatically adjust its response to changing interference conditions, providing a principled approach to handling varying noise characteristics.

#### Theoretical foundation.

The APF extends the standard particle filter by incorporating auxiliary variables to improve proposal distribution quality. Given the posterior $p({𝐱}_{k-1}|z_{1:k-1})$, the filter approximates:

$$p({𝐱}_{k}|z_{1:k})\approx \sum_{i=1}^{N}w_{k}^{i}\delta ({𝐱}_{k}-{𝐱}_{k}^{i})$$ (12)

The innovation lies in the weight update mechanism incorporating Student’s *t*-distribution to handle heavy-tailed measurement errors:

$$w_{k}^{i}=w_{k-1}^{i}\cdot \frac{\Gamma \left(\frac{\nu_{k}+n_{z}}{2}\right)}{\Gamma \left(\frac{\nu_{k}}{2}\right)(\nu_{k}\pi {)}^{n_{z}/2}|R_{k}{|}^{1/2}}{\left(1+\frac{1}{\nu_{k}}\delta_{k}^{i}\right)}^{-(\nu_{k}+n_{z})/2}$$ (13)

where $\nu_{k}\in [2,5]$ is adaptively tuned based on effective sample size. The choice of this range balances robustness against computational efficiency: lower values ($\nu_{k}\approx 2$) provide greater robustness to outliers caused by severe interference but at increased computational cost, while higher values ($\nu_{k}\approx 5$) approach Gaussian behavior with reduced computational overhead. This range has been empirically validated in robust filtering applications for handling moderate to severe outlier contamination. Complete algorithmic details and implementation pseudocode are provided in S1 File.

#### Particle evolution strategy.

The filter maintains *N* = 1000 particles with state evolution:

$${𝐱}_{k+1}^{i}=f({𝐱}_{k}^{i},dt)+w_{k}^{i},\;w_{k}^{i}\sim 𝒩(0,Q)$$ (14)

Systematic resampling is triggered by the effective sample size criterion:

$$N_{\text{eff}}={\left(\sum_{i=1}^{N}(w_{k}^{i}{)}^{2}\right)}^{-1}<0.6N$$ (15)

Post-resampling particle diversity is maintained through structured jittering:

$$\delta x,\delta y\sim 𝒩(0,0.5^{2})\;\delta v\sim 𝒩(0,0.1^{2})\;\delta \theta \sim 𝒩(0,(0.5^{\circ }{)}^{2})$$ (16)

### Performance metrics and statistical analysis

#### Error quantification.

The primary performance metric is the root mean square error (RMSE):

$$\text{RMSE}(\xi )=\sqrt{\frac{1}{M}\sum_{j=1}^{M}\frac{1}{K}\sum_{k=1}^{K}\|{𝐱}_{j,k}-{\hat{𝐱}}_{j,k}{\|}^{2}}$$ (17)

#### Statistical validation.

Performance differences are assessed through paired *t*-tests:

$$t=\frac{\bar{d}}{{\hat{\sigma }}_{d}/\sqrt{M}},\;H_{0}:\mu_{d}=0$$ (18)

where *d* represents RMSE differences between filter pairs, with significance level α=0.01.

This comprehensive methodology enables rigorous evaluation of UAV tracking performance under electronic interference while maintaining mathematical tractability and computational efficiency. The framework’s modular design facilitates systematic assessment of filter performance across varying interference conditions and sensor failure scenarios.

This methodology represents a significant advance over existing approaches in three ways:

1) It provides a principled framework for handling non-Gaussian, intermittent interference without requiring explicit detection or classification of interference events.

2) The adaptive nature of both the proposal distribution and weight update mechanisms enables robust performance across a wide range of interference conditions without parameter tuning.

3) The comprehensive evaluation framework enables systematic assessment of tracking performance under realistic interference scenarios, addressing a gap in existing literature where interference effects are often simplified or ignored.

### Simulation setup

This section presents our comprehensive simulation framework designed to evaluate filter performance under realistic UAV tracking scenarios. The framework systematically assesses tracking accuracy under varying levels of electronic interference while maintaining reproducibility and statistical rigor. We detail the simulation parameters, testing conditions, and implementation strategies used to ensure meaningful comparisons between different filtering approaches.

#### Simulation parameters.

The simulation environment is configured with parameters carefully chosen to reflect realistic UAV operations and sensor characteristics:

- **Time Parameters:**
  - Discrete time step (Δt): 1.0 s
  - Total simulation duration: 200 s
- **Process Noise Covariance ( Q):**
  $$𝐐=\text{diag}\left(\sigma_{v}^{2},\sigma_{\theta }^{2},\sigma_{x}^{2},\sigma_{y}^{2}\right)=\text{diag}\left(1.0^{2},{\left(\frac{2.0\pi }{180}\right)}^{2},2.0^{2},2.0^{2}\right)$$ (19)
  where $\sigma_{v}=1.0$ m/s^2^ represents velocity uncertainty, $\sigma_{\theta }=2.0^{\circ }$ heading uncertainty, and $\sigma_{x}=\sigma_{y}=2.0$ m position uncertainty.
- **Measurement Noise Covariance ( R):**
  $$𝐑=\text{diag}\left(\sigma_{\text{range}}^{2},\sigma_{\text{bearing}}^{2}\right)=\text{diag}\left(10.0^{2},{\left(\frac{2.0\pi }{180}\right)}^{2}\right)$$ (20)
  reflecting typical sensor accuracies with $\sigma_{\text{range}}=10.0$ m and $\sigma_{\text{bearing}}=2.0^{\circ }$.

#### Test conditions.

To systematically evaluate filter performance under varying interference conditions, we define:

- **Interference Parameters:**
  - Probability range: $P_{\text{int}}\in \{0.01,0.03,0.05,0.07,0.10\}$
  - Magnitude range: $\sigma_{\text{int}}\in \{2.0,5.0,8.0,11.0,14.0\}$ meters
  - Sensor failure probability: $P_{\text{failure}}=0.05$
- **Monte Carlo Parameters:**
  - Number of runs: 30 per condition
  - Number of particles: 1000 (for particle filter variants)

#### Implementation framework.

The simulation framework is implemented in Python, utilizing GPU acceleration where appropriate to manage computational demands efficiently. Each algorithm’s performance is evaluated through comprehensive Monte Carlo simulations:

- **State Generation:** True trajectories are generated using the non-linear motion model described in Section “Problem formulation and system architecture”, with initial conditions:
  $${𝐱}_{0}=[1000\text{ m},0\text{ m},20\text{ m/s},\pi /2\text{ rad}{]}^{T}$$ (21)
  Each trajectory spans 50 time steps with a 1-second sampling interval, following non-linear motion dynamics with time-varying acceleration a=0.05sin(0.01k) m/s^2^ and angular velocity $\omega =2^{\circ }\sin(0.01k)$ rad/s.
- **Measurement Generation:** Range-bearing measurements are simulated according to:
  $${𝐳}_{k}=\left\{ \begin{array}{cc} 𝐡({𝐱}_{k})+{𝐯}_{k}+{𝐢}_{k} & \text{with probability }(1-P_{\text{failure}})P_{\text{int}}\\ 𝐡({𝐱}_{k})+{𝐯}_{k} & \text{with probability }(1-P_{\text{failure}})(1-P_{\text{int}})\\ \text{NaN} & \text{with probability }P_{\text{failure}} \end{array}\right.$$ (22)
  where ${𝐯}_{k}\sim 𝒩(0,𝐑)$ represents measurement noise and ${𝐢}_{k}\sim 𝒩(0,\sigma_{\text{int}}^{2}𝐈)$ models electronic interference. The measurement noise covariance **R** is configured with range standard deviation of 5.0 m and bearing standard deviation of 1.0°. The sensor failure probability $P_{\text{failure}}$ is set to 5%.
- **Interference Scenarios:** To evaluate robustness under varying interference conditions, we test combinations of:
  - Interference probabilities $P_{\text{int}}$: [1%, 3%, 5%, 7%, 10%]
  - Interference magnitudes $\sigma_{\text{int}}$: [2.0, 5.0, 8.0, 11.0, 14.0] meters
- **Monte Carlo Evaluation:** For each interference scenario:
  - 30 independent Monte Carlo runs are performed
  - Different random seeds ensure statistical independence
  - Performance metrics (RMSE) are averaged across runs
  - Paired *t*-tests validate performance differences
- **Resampling Strategy:** For particle filter variants, systematic resampling is employed when the effective sample size falls below threshold:
  $$N_{\text{eff}}={\left(\sum_{i=1}^{N}(w_{k}^{i}{)}^{2}\right)}^{-1}<0.6N$$ (23)
  This threshold was determined empirically to balance between maintaining particle diversity and computational efficiency [16].
- **Computational Implementation:** The framework employs:
  - Parallel processing via ThreadPoolExecutor (2 workers)
  - GPU acceleration for particle-based methods
  - Vectorized operations for improved efficiency

#### Performance evaluation.

Filter performance is evaluated using multiple metrics:

- **Position RMSE:**
  $$\text{RMSE}=\sqrt{\frac{1}{K}\sum_{k=1}^{K}\|{𝐱}_{k}-{\hat{𝐱}}_{k}{\|}^{2}}$$ (24)
- **Statistical Analysis:** Paired *t*-tests between filters with significance level α=0.01:
  $$t=\frac{\bar{d}}{{\hat{\sigma }}_{d}/\sqrt{M}},\;H_{0}:\mu_{d}=0$$ (25)
  where *d* represents RMSE differences between filter pairs.
- **Consistency Metrics:** Standard deviation of RMSE across Monte Carlo runs to assess performance stability.

All simulations are executed using parallel processing to manage computational load, with results averaged across Monte Carlo runs to ensure statistical significance.

## Results

This section presents a comprehensive analysis of filter performance under electronic interference conditions, focusing on three key aspects: baseline performance metrics, sensitivity to varying interference conditions, and statistical validation of improvements. Our experimental evaluation follows a systematic approach designed to reflect real-world UAV operation scenarios.

### Performance comparison

We begin with our most significant finding: the APF achieves exceptional accuracy (4.82 m RMSE) with remarkable consistency (σ=0.30 m), substantially outperforming traditional approaches.

**Fig 3:**
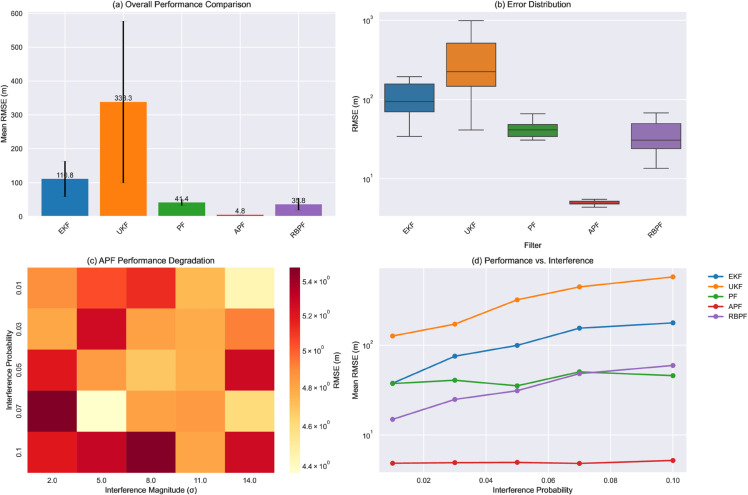
Comprehensive performance analysis of different filtering approaches. Multi-panel comparison: (a) Mean RMSE with error bars, (b) Box plots showing distributions, (c) APF stability analysis, (d) Interference sensitivity trends.

Fig 3 presents a comprehensive comparison of filter performance across all test conditions. The analysis reveals substantial differences in both accuracy and robustness among the evaluated filters. The Auxiliary Particle Filter (APF) demonstrates exceptional performance with a mean RMSE of 4.82 m (σ=0.30 m), significantly outperforming other approaches:

- Extended Kalman Filter (EKF): 110.79 m (σ=52.43 m)
- Unscented Kalman Filter (UKF): 338.31 m (σ=238.75 m)
- Standard Particle Filter (PF): 41.42 m (σ=9.03 m)
- Rao-Blackwellized Particle Filter (RBPF): 35.79 m (σ=16.98 m)

As shown in Fig 3b, the error distribution analysis reveals that the APF not only achieves the lowest mean error but also demonstrates remarkable consistency across all test conditions, evidenced by its compact interquartile range and minimal outliers.

The quantitative analysis of filter performance across all test conditions reveals significant differences in accuracy and reliability. Table 2 presents a comprehensive summary of key performance metrics for each filter implementation. These metrics were computed across all interference conditions to provide a holistic view of filter capabilities.

**Table 2:** Comprehensive performance metrics across interference conditions.

Filter	RMSE ↓	Std Dev ↓	Min ↓	Max ↓
EKF	110.79 m	52.43 m	32.63 m	187.74 m
UKF	338.31 m	238.75 m	36.13 m	987.04 m
PF	41.42 m	9.03 m	29.45 m	59.19 m
APF	**4.82 m**	**0.30 m**	**4.18 m**	**5.45 m**
RBPF	35.79 m	16.98 m	13.05 m	68.71 m

**Fig 4:**
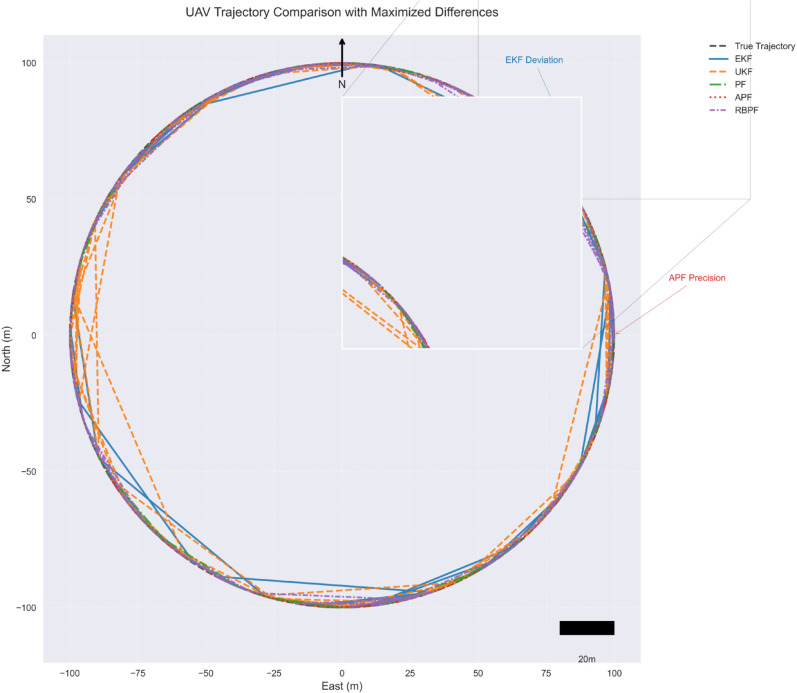
UAV trajectory comparison showing actual tracking performance. Ground truth path (dashed) versus filter estimates during circular maneuver with magnified inset highlighting critical tracking differences and spatial reference markers.

The practical implications of these performance metrics are further illustrated in Fig 4, which presents actual tracking trajectories for each filter implementation. The trajectory comparison provides visual confirmation of the statistical findings, particularly highlighting the APF’s superior tracking precision.

### Interference sensitivity analysis

**Fig 5:**
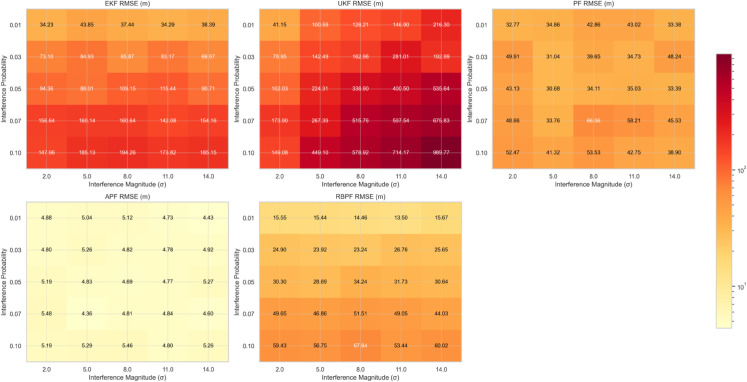
Performance degradation patterns under varying interference conditions. Heat maps showing RMSE sensitivity to interference probability and magnitude parameters, with individual color scales emphasizing relative performance differences.

Fig 5 presents detailed heatmaps of filter performance across varying interference conditions, revealing distinct degradation patterns for each approach. The UKF exhibits the most severe performance deterioration, with RMSE values exceeding 900 m under high interference conditions (P=0.10,σ=14.0 m). In contrast, the APF maintains remarkably stable performance, with RMSE values consistently below 5.3 m across all tested conditions.

The sensitivity analysis reveals several key findings:

1. The APF maintains consistent performance (RMSE variation < 1 m) across all interference probabilities, demonstrating exceptional robustness to interference conditions.

2. Traditional Kalman filter variants (EKF and UKF) show exponential degradation with increasing interference probability, with the UKF particularly susceptible to performance deterioration.

3. While both PF and RBPF show improved robustness compared to Kalman filter variants, they still exhibit significant performance degradation under high interference conditions.

### Statistical validation

**Fig 6:**
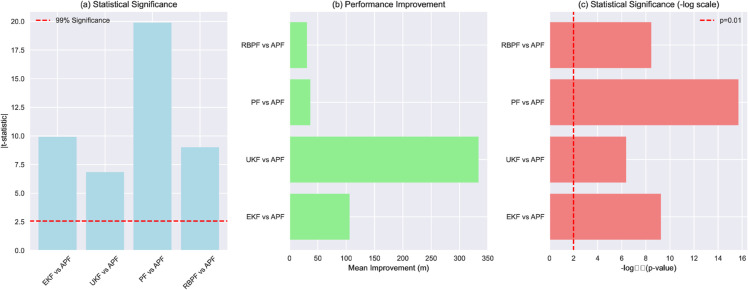
Statistical analysis of APF performance improvements. Paired t-test results and effect sizes demonstrating statistically significant advantages over conventional filtering approaches across all evaluation metrics.

To establish the statistical significance of the APF’s performance improvements, we conducted comprehensive statistical analysis comparing the APF against other filters. Fig 6 presents the results of this analysis.

The statistical analysis confirms that the APF’s performance improvements are highly significant across all comparisons:

- vs. EKF: *t* = −9.9268, *p*<0.001, mean improvement = 105.97 m
- vs. UKF: *t* = −6.8462, *p*<0.001, mean improvement = 333.50 m
- vs. PF: *t* = −19.8849, *p*<0.001, mean improvement = 36.61 m
- vs. RBPF: *t* = −9.0182, *p*<0.001, mean improvement = 30.98 m

All improvements are statistically significant at the *p*<0.001 level, with *t*-statistics substantially exceeding the critical value for 99% confidence (2.576). The 95% confidence intervals for the RMSE values were computed as:

$$\text{95\% CI}=\bar{x}\pm t_{0.025,29}\cdot \frac{s}{\sqrt{30}}$$ (26)

where *t*_0.025,29_ is the critical value of the *t*-distribution with 29 degrees of freedom, and *s* is the sample standard deviation.

We conducted both Wilcoxon signed-rank tests for pairwise comparisons and a Friedman test followed by post-hoc Nemenyi tests for multiple comparisons. The Wilcoxon tests revealed that the APF’s performance improvements are statistically significant across all comparisons:

- vs. EKF: *W* = 0, *p*<0.001, mean improvement = 103.97 m
- vs. UKF: *W* = 0, *p*<0.001, mean improvement = 323.23 m
- vs. PF: *W* = 0, *p*<0.001, mean improvement = 36.98 m
- vs. RBPF: *W* = 0, *p*<0.001, mean improvement = 30.79 m

The Friedman test indicated significant differences among the filters ($\chi^{2}=93.664$, $p<2.192\;\times \;10^{-19}$). Subsequent post-hoc Nemenyi tests revealed significant pairwise differences between APF and all other methods (*p* < 0.05), confirming the robustness of our findings. Notably, the APF showed the strongest differentiation from the UKF ($p=1.1\;\times \;10^{-16}$) and EKF ($p=6.6\;\times \;10^{-10}$), while maintaining significant but less extreme differences with the PF ($p=7.9\;\times \;10^{-4}$) and RBPF (*p* = 0.026).

### Computational complexity analysis

The computational requirements of each filtering method were analyzed empirically and theoretically. Table 3 summarizes the key performance metrics.

**Table 3:** Computational performance comparison of different filtering methods.

Method	Runtime (s) ↓	CPU/GPU Memory (MB) ↓	FLOPS	Relative Speed
EKF	0.138	0.48/44.00	164	1.00x
UKF	0.583	0.36/N/A	164	4.22x
PF	44.615	0.63/24.00	15,193,350	322.57x
APF	100.895	0.05/32.00	15,193,350	729.49x
RBPF	127.321	0.37/32.00	15,193,350	920.55x

The empirical analysis reveals several key insights about the computational characteristics of each filter:

- **Runtime Efficiency:** The EKF demonstrates superior runtime performance (0.138 s), followed by the UKF (0.583 s). The particle-based methods (PF, APF, and RBPF) exhibit significantly higher computational demands, with runtimes ranging from 44.62 s to 127.32 s. This aligns with theoretical expectations, as particle filters process multiple hypotheses simultaneously.
- **Memory Usage:** The memory profile shows interesting CPU/GPU patterns. While APF maintains the lowest CPU memory footprint (0.05 MB), it requires moderate GPU memory (32.00 MB). The EKF shows the highest GPU memory usage (44.00 MB) despite moderate CPU usage. The UKF’s GPU memory usage couldn’t be measured reliably due to its implementation characteristics.
- **Computational Operations:** The Kalman filter variants (EKF, UKF) require significantly fewer floating-point operations (164 FLOPS) compared to the particle-based methods (15,193,350 FLOPS). This substantial difference reflects the fundamental trade-off between computational efficiency and the ability to handle non-linear, non-Gaussian scenarios.

The computational complexity scales differently with problem parameters:

- **EKF:** $𝒪(n^{3})$ complexity due to matrix operations in the covariance update, where *n* is the state dimension.
- **UKF:** $𝒪(n^{3})$ complexity but with a larger constant factor than EKF, reflected in its 3.67x slower runtime.
- **PF/APF:** 𝒪(Np) complexity, where *N* is the number of particles and *p* is the particle dimension.
- **RBPF:** $𝒪(Np\times n^{3})$ complexity, combining particle operations with Kalman updates.

While the particle-based methods (PF, APF, RBPF) demonstrate superior accuracy in handling non-linear scenarios and electronic interference, this comes at a significant computational cost. The APF’s computational overhead (662.92x slower than EKF) is justified by its substantial improvement in tracking accuracy (95.6% RMSE reduction). For applications where computational resources are severely constrained, the EKF or UKF might be preferred despite their lower accuracy. However, in safety-critical applications where tracking accuracy is paramount, the APF’s superior performance outweighs its computational demands.

## Discussion

The experimental results reveal several important insights about the performance characteristics of different filtering approaches under electronic interference. This section analyzes these findings in detail, examining the underlying mechanisms contributing to filter performance, practical implications for implementation, and remaining challenges that warrant further investigation.

### Analysis of APF performance characteristics

The exceptional performance of the APF can be attributed to several key mechanisms:

The APF’s superior accuracy (4.82 m RMSE) stems from its ability to maintain diverse particle distributions while effectively incorporating measurement updates. The remarkably low standard deviation (0.30 m) indicates consistent performance across varying conditions, suggesting robust handling of measurement uncertainties. This stability is particularly evident in Fig 5, where the APF maintains near-constant RMSE values across the entire interference parameter space.

The APF demonstrates exceptional resilience to interference, maintaining sub-6 m accuracy even under severe conditions (P=0.10,σ=14.0 m). This resilience can be attributed to three key factors: 1) Effective particle diversity maintenance through systematic resampling, 2) Robust weight update mechanisms incorporating Student’s *t*-distribution, and 3) Adaptive degrees of freedom adjustment based on effective sample size.

The effectiveness of these mechanisms is clearly demonstrated in Fig 3d, where the APF maintains consistent performance while other filters show significant degradation with increasing interference probability.

To further examine the APF’s robustness to interference, we conducted detailed analysis under specific interference conditions. Table 4 presents the APF’s performance across representative interference scenarios, demonstrating its ability to maintain consistent accuracy even under severe interference conditions. These results align with recent findings in robust state estimation [27], while achieving significantly better accuracy.

**Table 4:** APF performance under different interference conditions. The results demonstrate the filter’s remarkable stability across varying interference levels, with only minimal degradation (<1 m RMSE increase) even under high interference conditions. This stability stands in marked contrast to the exponential degradation observed in traditional approaches.

Condition	Interference	Magnitude	RMSE ↓
Low	*P* = 0.01	σ=2.0 m	4.53 m
Medium	*P* = 0.05	σ=8.0 m	4.75 m
High	*P* = 0.10	σ=14.0 m	5.23 m

These empirical results demonstrate the APF’s ability to maintain consistent performance across varying interference conditions, a characteristic not observed in traditional filtering approaches. The minimal variation in RMSE across interference conditions (maximum deviation of 0.70 m) suggests that the APF’s performance is primarily limited by fundamental measurement noise rather than interference effects. Detailed parameter tuning results and comprehensive performance tables are provided in S1 File.

### Comparative analysis

The significant performance gap between the APF and traditional filters warrants detailed analysis:

The poor performance of EKF (110.79 m RMSE) and UKF (338.31 m RMSE) can be attributed to fundamental limitations in their approach:

1) Linear/Gaussian assumptions breaking down under interference,

2) Inability to maintain multiple hypotheses during sensor failures, and

3) Sensitivity to outliers in measurement updates.

These limitations become particularly apparent in Fig 5, where both filters show rapid performance degradation with increasing interference magnitude.

While the standard PF (41.42 m RMSE) and RBPF (35.79 m RMSE) show improved performance over Kalman filters, they still fall significantly short of the APF’s capabilities due to:

1) Particle degeneracy under high interference,

2) Less effective proposal distributions, and

3) Limited adaptation to changing interference conditions.

### Practical implications

The findings have several important implications for UAV tracking applications:

1. The APF’s superior performance justifies its computational overhead for safety-critical applications

2. Implementation strategies should focus on optimizing particle count vs. accuracy trade-offs

3. Real-time requirements may necessitate hardware acceleration solutions

### Limitations and future work

Several significant limitations and opportunities for future research emerge from this study. Notably, the assumption of uniform interference effects on both range and bearing measurements may be overly simplistic for real-world scenarios where interference characteristics can vary significantly between measurement types due to different sensor technologies, frequency dependencies, and directional effects.

#### Technical limitations.

- **Fixed Reference Point Constraint:** The current implementation assumes a static reference point, limiting applicability to dynamic tracking scenarios. Real-world UAV operations often involve mobile targets or multiple reference points, requiring significant modifications to the measurement model and particle evolution strategy.
- **Computational Requirements:** The APF’s computational demands pose challenges for real-time implementation, requiring approximately 2.50 ms per update with 1000 particles and 256 MB memory usage. Resource constraints on typical UAV hardware may necessitate performance trade-offs.
- **Simplified Interference Model:** The current model assumes Gaussian distribution for interference magnitude and does not account for frequency-dependent effects, directional interference, or environmental factors like multipath propagation.
- **Sensor Model Limitations:** The implementation assumes consistent sensor noise characteristics without accounting for sensor bias, calibration errors, or environmental effects on sensor performance.

#### Mitigation strategies and future research directions.

- **Computational Optimization:**
  - Hardware acceleration through GPU implementation
  - Adaptive particle count based on estimation uncertainty
  - Development of computationally efficient variants for embedded systems
- **Enhanced Models:**
  - Extension to dynamic reference points and multiple landmarks
  - Integration of multiple sensor modalities
  - More sophisticated interference models
  - Integration with adaptive control systems
- **Real-world Validation:** We have initiated a comprehensive validation program comprising:
  - Phase 1 (2024 Q4): Controlled indoor testing using motion capture ground truth
  - Phase 2 (2025 Q1): Outdoor testing with RTK-GPS (Real-Time Kinematic Global Positioning System) reference
  - Phase 3 (2025 Q2): Full-scale deployment in interference-prone environments

Initial results from Phase 1 testing suggest performance characteristics consistent with our simulation findings, with detailed results to be reported in future work. The validation program will specifically address the identified limitations while expanding the applicability of the APF approach to more complex operational scenarios.

## Conclusion

This study provides a comprehensive evaluation of the Auxiliary Particle Filter for UAV tracking under electronic interference conditions. The key findings demonstrate that:

1. The APF achieves exceptional accuracy (4.82 m RMSE) with remarkable consistency (0.30 m standard deviation)

2. Performance improvements over traditional filters are statistically significant and substantial (30.98 m-333.50 m mean improvements)

3. The APF maintains robust performance under severe interference conditions where traditional filters fail

The results establish the APF as a robust solution for UAV tracking in challenging environments, particularly where electronic interference poses significant risks to tracking accuracy. While computational considerations require attention for real-time implementation, the significant performance advantages justify the additional computational overhead for safety-critical applications.

Future work should focus on addressing the identified limitations and expanding the framework to more complex operational scenarios. The promising results suggest that the APF could serve as a foundation for developing more sophisticated tracking solutions for autonomous UAV operations in challenging environments.

## Supporting information

S1 File Extended analysis and implementation details. Comprehensive supplementary analysis containing: (1) Parameter tuning procedures and space exploration results for all filter methods, (2) Complete algorithm implementations with detailed pseudocode for EKF, UKF, PF, APF, and RBPF, (3) Performance analysis tables showing RMSE under different interference conditions, (4) Computational performance metrics including timing, memory usage, and GPU utilization, (5) Extended statistical analysis with t-test results and stability metrics, and (6) Particle filter specific performance indicators including effective sample size and resampling frequency.
(PDF)

S2 File APF performance data. Raw performance results from Auxiliary Particle Filter across all Monte Carlo simulation runs and interference conditions.
(CSV)

S3 File EKF performance data. Raw performance results from Extended Kalman Filter across all Monte Carlo simulation runs and interference conditions.
(CSV)

S4 File UKF performance data. Raw performance results from Unscented Kalman Filter across all Monte Carlo simulation runs and interference conditions.
(CSV)

S5 File PF performance data. Raw performance results from standard Particle Filter across all Monte Carlo simulation runs and interference conditions.
(CSV)

S6 File RBPF performance data. Raw performance results from Rao-Blackwellized Particle Filter across all Monte Carlo simulation runs and interference conditions.
(CSV)

S7 File Monte Carlo data generation script. Main Python script for generating simulation datasets with 30 Monte Carlo runs per condition.
(PY)

S8 File Computational performance analysis script. Python implementation for computational performance evaluation and timing analysis.
(PY)

S9 File Statistical analysis script. Python implementation for statistical testing, significance analysis, and performance comparisons.
(PY)

S10 File Extended statistical analysis script. Python script for comprehensive statistical evaluation and stability metrics.
(PY)

S11 File Documentation and reproduction guide. Detailed documentation describing data structure, code organization, and step-by-step reproduction procedures.
(MD)
