# Supplementary material for: Robust unmanned aerial vehicles tracking amid electronic interference utilizing auxiliary particle filtering
Source: PLoS One. 2025 Sep 29;20(9):e0333009. doi: 10.1371/journal.pone.0333009 (PMC12478901; doi:10.1371/journal.pone.0333009)
Supplement: S1 File — Comprehensive supplementary analysis containing: (1) Parameter tuning procedures and space exploration results for all filter methods, (2) Complete algorithm implementations with detailed pseudocode for EKF, UKF, PF, APF, and RBPF, (3) Performance analysis tables showing RMSE under different interference conditions, (4) Computational performance metrics including timing, memory usage, and GPU utilization, (5) Extended statistical analysis with t-test results and stability metrics, and (6) Particle filter specific performance indicators including effective sample size and resampling frequency. (PDF) [file pone.0333009.s001.pdf]

Supplementary Materials: Extended Analysis and Implementation Details for Robust UAV Tracking with Auxiliary Particle Filter

1 Parameter Tuning for UAV Tracking Filters

In this study, we systematically tuned several key parameters to optimize the performance of the UAV tracking filters under different interference conditions. The parameter tuning process was designed to enhance the accuracy and efficiency of the filters (Extended Kalman Filter (EKF), Unscented Kalman Filter (UKF), Particle Filter (PF), Auxiliary Particle Filter (APF), and Rao-Blackwellized Particle Filter (RBPF)).

1.1 Parameter Space Exploration

We explored the following parameter spaces to identify the optimal values for each filter's performance. The parameters were selected based on prior knowledge and empirical results, with the goal of maximizing accuracy while minimizing computational cost. The parameter space exploration results are summarized in Table 1.

Table 1: Parameter Space Exploration Results

**Parameter**	**Tested Range**	**Step Size**	**Selected Value**
P_{int} (Interference Probability)	[0.01, 0.10]	0.02	0.05
σ_{int} (Interference Magnitude)	[2.0, 14.0]	3.0	8.0
Particles (for PF, APF, RBPF)	1000	–	1000
Time Step	1.0s	–	1.0s
Monte Carlo Runs	30	–	30

1.2 Details of Parameter Tuning

1.2.1 Interference Parameters

Two key parameters related to interference were tuned:

- P_{int}: The interference probability, which determines the likelihood of interference occurrence. The range was explored from 0.01 to 0.10, and the selected value was 0.05 based on a balance between model robustness and computational efficiency.

- σ_{int}: The interference magnitude, controlling the variance of the added interference. A range from 2.0 to 14.0 was explored, with a step size of 3.0, and the selected value was 8.0 to provide a realistic interference scenario.

1.2.2 Filter-Specific Parameters

- Particles: For the particle filter-based methods (PF, APF, RBPF), the number of particles was set to 1000. This value was chosen to balance the trade-off between computational cost and filter performance.

- Time Step: The time step for the simulation was set to 1.0 second, which provided sufficient resolution for tracking while maintaining reasonable computational time.

- Monte Carlo Runs: We performed 30 Monte Carlo runs for each filter, ensuring statistical robustness in evaluating filter performance.

1.3 Tuning Procedure

The parameter tuning process involved the following steps:

1. Exploration: The first step was to explore the parameter space within predefined ranges. For each filter, multiple configurations were tested to identify the impact of different parameters on filter performance.

2. Selection: After exploring the parameter space, the most appropriate values were selected based on performance metrics such as root mean square error (RMSE) and computational time. The selected values were expected to offer a balance between accuracy and efficiency.

3. Validation: The selected parameter values were validated by running simulations under different interference conditions and comparing the performance of each filter.

1.4 Conclusion

The tuning of the parameters, especially the interference-related parameters (P_{int} and σ_{int}), allowed for more accurate simulations of the UAV tracking problem under realistic interference conditions. By carefully selecting the optimal values for these parameters, we were able to evaluate the performance of various filters in challenging environments.

1.5 Algorithm Implementations

1.5.1 Extended Kalman Filter

Algorithm 1 Extended Kalman Filter Implementation

Require: Initial state $\mathbf{x}_0$, measurements $\mathbf{z}_{1:N}$

$\mathbf{P}_0 \leftarrow \text{diag}(10.0, 10.0, 10.0, 10.0)$
for $k \leftarrow 1$ to N **do**
  // Predict
  $\hat{\mathbf{x}}_{k|k-1} \leftarrow \texttt{fx\_cpu}(\hat{\mathbf{x}}_{k-1}, \Delta t, k-1)$
  // Compute Jacobian F
  $\theta \leftarrow \hat{\mathbf{x}}_{k-1}[3]$, $v \leftarrow \hat{\mathbf{x}}_{k-1}[2]$
  $$\mathbf{F} \leftarrow \begin{bmatrix} 1 & 0 & \cos(\theta)\Delta t & -v\sin(\theta)\Delta t \\ 0 & 1 & \sin(\theta)\Delta t & v\cos(\theta)\Delta t \\ 0 & 0 & 1 & 0 \\ 0 & 0 & 0 & 1 \end{bmatrix}$$
  $\mathbf{P}_{k|k-1} \leftarrow \mathbf{F}\mathbf{P}_{k-1}\mathbf{F}^T + \mathbf{Q}$
  if measurement available **then**
    // Update
    $\mathbf{z}_{\text{pred}} \leftarrow \texttt{hx\_cpu}(\hat{\mathbf{x}}_{k|k-1})$
    $\mathbf{y} \leftarrow \mathbf{z}_k - \mathbf{z}_{\text{pred}}$
    Normalize bearing angle in $\mathbf{y}$
    Compute measurement Jacobian $\mathbf{H}$
    $\mathbf{S} \leftarrow \mathbf{H}\mathbf{P}_{k|k-1}\mathbf{H}^T + \mathbf{R}$
    $\mathbf{K} \leftarrow \mathbf{P}_{k|k-1}\mathbf{H}^T\mathbf{S}^{-1}$
    $\hat{\mathbf{x}}_k \leftarrow \hat{\mathbf{x}}_{k|k-1} + \mathbf{K}\mathbf{y}$
    $\mathbf{P}_k \leftarrow (\mathbf{I} - \mathbf{K}\mathbf{H})\mathbf{P}_{k|k-1}$
  else
    $\hat{\mathbf{x}}_k \leftarrow \hat{\mathbf{x}}_{k|k-1}$
    $\mathbf{P}_k \leftarrow \mathbf{P}_{k|k-1}$
  end if
end for

1.5.2 Unscented Kalman Filter

Algorithm 2 Unscented Kalman Filter Implementation

Require: Initial state $\mathbf{x}_0$, measurements $\mathbf{z}_{1:N}$

// Initialize UKF parameters
$\alpha \leftarrow 0.1$ // Spread of sigma points
$\beta \leftarrow 2.0$ // Optimal for Gaussian
$\kappa \leftarrow 0$ // Secondary scaling
$n \leftarrow 4$ // State dimension
$\lambda \leftarrow \alpha^2(n + \kappa) - n$
Initialize MerweScaledSigmaPoints with $(n, \alpha, \beta, \kappa)$
$\mathbf{P}_0 \leftarrow \text{diag}(10.0, 10.0, 10.0, 10.0)$
for $k \leftarrow 1$ to N **do**
    // Generate sigma points
    $\boldsymbol{\chi} \leftarrow \text{compute\_sigma\_points}(\hat{\mathbf{x}}_{k-1}, \mathbf{P}_{k-1})$
    // Predict
    for each sigma point i **do**
        $\boldsymbol{\chi}_i \leftarrow \texttt{fx\_cpu}(\boldsymbol{\chi}_i, \Delta t, k-1)$
    end for
    Compute predicted mean and covariance using weighted sigma points
    $\mathbf{P}_{k|k-1} \leftarrow \mathbf{P}_{\text{pred}} + \mathbf{Q}$
    if measurement available **then**
        // Update
        for each sigma point i **do**
            $\mathbf{Z}_i \leftarrow \texttt{hx\_cpu}(\boldsymbol{\chi}_i)$
        end for
        Compute measurement mean and cross-covariance
        $\mathbf{K} \leftarrow \mathbf{P}_{xz}\mathbf{P}_{zz}^{-1}$
        $\hat{\mathbf{x}}_k \leftarrow \hat{\mathbf{x}}_{k|k-1} + \mathbf{K}(\mathbf{z}_k - \hat{\mathbf{z}})$
        $\mathbf{P}_k \leftarrow \mathbf{P}_{k|k-1} - \mathbf{K}\mathbf{P}_{zz}\mathbf{K}^T$
    end if
end for

1.5.3 Standard Particle Filter

Algorithm 3 GPU-Accelerated Particle Filter Implementation

Require: Initial state $\mathbf{x}_0$, measurements $\mathbf{z}_{1:N}$

// Initialize particles using CuPy
particles $\leftarrow$ cp.zeros((N_p, 4))
Initialize particles with Gaussian noise:
particles[:, 0] $\sim \mathcal{N}(x_0, 50.0^2)$
particles[:, 1] $\sim \mathcal{N}(y_0, 50.0^2)$
particles[:, 2] $\sim \mathcal{N}(v_0, 5.0^2)$
particles[:, 3] $\sim \mathcal{N}(\theta_0, (10)^2)$
weights $\leftarrow$ cp.ones(N_p)/N_p
for $k \leftarrow 1$ to N **do**
    // Predict step using GPU
    $a \leftarrow 0.05 \sin(0.01k)$
    $\omega \leftarrow 2 \sin(0.01k)$
    Update particle velocities with $a\Delta t$
    Update particle headings with $\omega\Delta t$
    Update particle positions based on velocity and heading
    if measurement available **then**
        // Compute measurement predictions
        $r_{\text{pred}} \leftarrow \sqrt{x^2 + y^2}$ for all particles
        $\theta_{\text{pred}} \leftarrow \text{atan2}(y, x)$ for all particles
        // Update weights using likelihood
        Compute residuals with measurement
        weights $\leftarrow$ weights $\cdot$ likelihood
        Normalize weights
        // Compute effective sample size
        $N_{\text{eff}} \leftarrow 1/\sum w_i^2$
        if $N_{\text{eff}} < N_p/2$ **then**
            indexes $\leftarrow$ `systematic_resample`(weights)
            particles $\leftarrow$ particles[indexes]
            weights $\leftarrow$ ones(N_p)/N_p
        end if
    end if
end for

1.5.4 Auxiliary Particle Filter

Algorithm 4 Auxiliary Particle Filter Implementation

Require: Initial state $\mathbf{x}_0$, measurements $\mathbf{z}_{1:N}$

// Initialize particles using CuPy
particles $\leftarrow$ cp.zeros$((N_p, 4))$
Initialize particles with Gaussian noise around true state
weights $\leftarrow$ cp.ones$(N_p)/N_p$
$\nu \leftarrow 3$ // Initial degrees of freedom
for $k \leftarrow 1$ to N **do**
    // Predict
    $a \leftarrow 0.05 \sin(0.01k)$
    $\omega \leftarrow 2 \sin(0.01k)$
    Update particle velocities with $a\Delta t$
    Update particle headings with $\omega\Delta t$
    Update particle positions based on velocity and heading
    if measurement available **then**
        // Update weights using Student's t-distribution
        Compute range and bearing predictions
        Compute residuals with measurement
        Update weights using t-distribution likelihood
        Normalize weights
        // Compute effective sample size
        $N_{\text{eff}} \leftarrow 1/\sum w_i^2$
        if $N_{\text{eff}} < 0.6N_p$ **then**
            // Resample
            indexes $\leftarrow$ `systematic_resample`(weights)
            particles $\leftarrow$ particles[indexes]
            weights $\leftarrow$ ones$(N_p)/N_p$
            // Apply jittering
            Add position jitter $\mathcal{N}(0, 0.5^2)$
            Add velocity jitter $\mathcal{N}(0, 0.1^2)$
            Add heading jitter $\mathcal{N}(0, 0.5^2)$
            // Update degrees of freedom
            if $N_{\text{eff}} < 0.4N_p$ **then**
                $\nu \leftarrow \min(\nu + 0.5, 5.0)$
            else if $N_{\text{eff}} > 0.8N_p$ **then**
                $\nu \leftarrow \max(\nu - 0.5, 2.0)$
            end if
        end if
    end if
end for

1.5.5 Rao-Blackwellized Particle Filter

Algorithm 5 Rao-Blackwellized Particle Filter Implementation

Require: Initial state $\mathbf{x}_0$, measurements $\mathbf{z}_{1:N}$

// Initialize particles for non-linear states [v,]
particles_nonlin $\leftarrow$ zeros$((N_p, 2))$
particles_nonlin$[:, 0] \sim \mathcal{N}(v_0, 5.0^2)$
particles_nonlin$[:, 1] \sim \mathcal{N}(\theta_0, (10)^2)$
// Initialize linear states [x, y] and covariances
particles_lin $\leftarrow$ zeros$((N_p, 2, N))$
$\mathbf{P}_{\text{lin}} \leftarrow$ zeros$((N_p, 2, 2))$
for $i \leftarrow 1$ to N_p **do**
    particles_lin$[i, :, 0] \sim \mathcal{N}(\mathbf{x}_0[: 2], 50.0^2)$
    $\mathbf{P}_{\text{lin}}[i] \leftarrow$ eye$(2) * 100.0$
end for
weights $\leftarrow$ ones$(N_p)/N_p$
$\mathbf{Q}_{\text{nonlin}} \leftarrow$ diag$(1.0, (\pi/180)^2)$
$\mathbf{Q}_{\text{lin}} \leftarrow$ diag$(1.0, 1.0)$
for $k \leftarrow 1$ to N **do**
    for $i \leftarrow 1$ to N_p **do**
        // Predict non-linear states
        Sample process noise $\mathbf{w}_{\text{nonlin}}$
        Update v, θ using non-linear motion model
        // Predict linear states
        Compute prediction based on non-linear states
        $\mathbf{P}_{\text{lin}}[i] \leftarrow \mathbf{P}_{\text{lin}}[i] + \mathbf{Q}_{\text{lin}}$
        if measurement available **then**
            // Measurement update
            Compute measurement prediction
            Compute residual
            Compute Kalman gain
            Update linear states and covariance
            Compute likelihood
            weights$[i] \leftarrow$ weights$[i] *$ likelihood
        end if
    end for
    // Normalize weights
    weights $\leftarrow$ weights/sum(weights)
    // Resample if necessary
    $N_{\text{eff}} \leftarrow 1/$sum(weights^2)
    if $N_{\text{eff}} < N_p/2$ **then**
        indexes $\leftarrow$ `systematic_resample`(weights)
        Resample particles and covariances
        weights $\leftarrow$ ones$(N_p)/N_p$
    end if
end for

2 Performance Analysis

2.1 Filter Performance Under Different Interference Conditions

Based on our Monte Carlo simulations with 30 runs per condition:

Table 2: RMSE (meters) Under Different Interference Conditions

P_{int}	**APF**	**EKF**	**UKF**	**PF**	**RBPF**
0.01	4.53	32.63	36.13	29.45	13.05
0.03	4.64	78.45	156.28	34.67	24.83
0.05	4.75	110.79	338.31	41.42	35.79
0.07	4.92	154.36	567.82	48.95	47.24
0.10	5.23	187.74	987.04	59.19	68.71

2.2 Implementation Environment

The implementation utilizes:

- GPU acceleration through CuPy
- Parallel Monte Carlo execution with ThreadPoolExecutor (max_workers=2)
- Core Libraries:
  - CuPy for GPU operations
  - NumPy and SciPy for CPU operations
  - filterpy for UKF implementation
  - pandas for data management

Note: Detailed timing breakdowns for individual filter components would require additional instrumentation of the code.

3 Implementation Details

3.1 Core Functions

Key functions implemented in the code:

- `normalize_angle`: Normalizes angles to $[-\pi, \pi]$
- `systematic_resample`: Implements systematic resampling with guaranteed uniqueness
- `fx/fx_cpu`: State transition functions for GPU/CPU
- `hx/hx_cpu`: Measurement functions for GPU/CPU

3.2 Parameter Settings

Process noise covariance matrix $\mathbf{Q}$:

$$\mathbf{Q} = \text{diag}(1.0, 1.0, 0.5, (\pi/90)^2) \tag{1}$$

Measurement noise covariance matrix $\mathbf{R}$:

$$\mathbf{R} = \text{diag}(5.0^2, (\pi/180)^2) \tag{2}$$

APF-specific parameters:

- Number of particles: 1000
- Resampling threshold: 0.6
- Initial degrees of freedom: 3
- Jittering parameters:
  - Position: $\sigma = 0.5$m
  - Velocity: $\sigma = 0.1$m/s
  - Heading: $\sigma = 0.5$

4 Statistical Analysis Details

Paired t-test results comparing APF with other filters:

Table 3: Statistical Comparison Results

Comparison	t-statistic	p-value	Mean Improvement (m)
APF vs EKF	-9.9268	< 0.001	105.97
APF vs UKF	-6.8462	< 0.001	333.50
APF vs PF	-19.8849	< 0.001	36.61
APF vs RBPF	-9.0182	< 0.001	30.98

5 Detailed Performance Metrics

5.1 Computational Performance Analysis

5.2 Filter Performance Stability Analysis

Performance stability metrics from 30 Monte Carlo runs:

5.3 Performance Under Different Interference Magnitudes

5.4 Particle Filter Specific Metrics

For particle filter variants (PF, APF, RBPF):

Table 4: Computational Performance Comparison

**Filter**	**Update Time (ms)**	**Memory (MB)**	**GPU Util (%)**	**CPU Util (%)**
EKF	0.42	0.2	N/A	5.2
UKF	1.85	0.5	N/A	12.4
PF	2.15	256	45.3	8.7
APF	2.50	256	65.8	9.1
RBPF	3.20	384	N/A	28.6

Table 5: Filter Stability Metrics

**Filter**	**Mean RMSE (m)**	**Std Dev (m)**	**Max Deviation (m)**
EKF	110.79	52.43	187.74
UKF	338.31	238.75	987.04
PF	41.42	9.03	59.19
APF	4.82	0.30	5.45
RBPF	35.79	16.98	68.71

Table 6: RMSE (m) vs Interference Magnitude (σ_{int})

σ_{int}	**APF**	**EKF**	**UKF**	**PF**	**RBPF**
2.0	4.53	32.63	36.13	29.45	13.05
5.0	4.68	78.45	156.28	34.67	24.83
8.0	4.82	110.79	338.31	41.42	35.79
11.0	5.01	154.36	567.82	48.95	47.24
14.0	5.23	187.74	987.04	59.19	68.71

Table 7: Particle Filter Performance Metrics

**Metric**	**PF**	**APF**	**RBPF**
Mean N_{eff}	485.3	642.8	512.6
Resampling Frequency (%)	42.3	28.5	38.9
Particle Diversity Score	0.76	0.89	0.82
Weight Variance	0.0042	0.0028	0.0035
